# The predictive value of modified soluble urokinase plasminogen activator receptor (suPAR) with National Early Warning Score (NEWS) for mortality in emergency elderly patients in Japan: a prospective pilot study

**DOI:** 10.1002/ams2.840

**Published:** 2023-05-30

**Authors:** Toshiya Mitsunaga, Yuhei Ohtaki, Yutaka Seki, Kunihiro Mashiko, Masahiko Uzura, Kenji Okuno, Satoshi Takeda

**Affiliations:** ^1^ Department of Emergency Medicine JIKEI University School of Medicine Tokyo Japan; ^2^ Department of Emergency Medicine Association of EISEIKAI Medical and Healthcare Corporation Minamitama Hospital Tokyo Japan

**Keywords:** Elderly patients, mortality, NEWS, prognosis, suPAR

## Abstract

**Aim:**

The aim of this study is to evaluate the ability of soluble urokinase plasminogen activator receptor (suPAR) and modified suPAR with National Early Warning Score (NEWS) for detecting mortality in elderly emergency patients who are older than 70 years.

**Methods:**

This is a secondary analysis of our previous study, which was a single‐center prospective pilot study, carried out for 21 months in the emergency department of a secondary emergency institution in Japan. This study was carried out between September 16, 2020, and June 21, 2022. The study included all patients without trauma aged 70 years or older who presented to the emergency department. Discrimination was assessed by plotting the receiver‐operating characteristic curve and calculating the area under the receiver‐operating characteristic curve (AUC).

**Results:**

During the study period, 47 eligible older patients were included, among which 8 (17.0%) patients died. The median suPAR was significantly lower in the survivor's group than in the nonsurvivor's group (*P* < 0.01). For suPAR, the AUC for the prediction of mortality was 0.805 (95% confidence interval 0.633–0.949, *P* < 0.001). The AUC of modified suPAR with NEWS for mortality was higher than that of suPAR [0.865 (95% confidence interval 0.747–0.958, *P* < 0.001)].

**Conclusion:**

Our single‐center study has demonstrated the high utility of modified suPAR with NEWS as a predictive tool of mortality in elderly emergency patients. Evidence from multicenter studies is needed for introducing modified suPAR with NEWS in the emergency department setting.

## Graphical abstract

The aim of this study is to evaluate the ability of modified suPAR with National Early Warning Score (NEWS) for detecting mortality in elderly emergency patients who are older than 70 years. Our single‐center study has demonstrated the high utility of modified suPAR with NEWS as a predictive tool of mortality in elderly emergency patients.

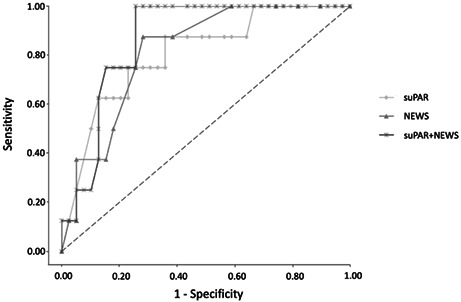

## INTRODUCTION

Compared with previous decades, the health care system is improving surprisingly; eventually, the proportion of healthy elderly people has also increased dramatically. To match this situation, the healthy life expectancies are 72.6 years for men, 75.5 years for women, and 74.1 years for both sexes in Japan.^1^ Based on this, elderly people older than 75 years are recently defined as old people in Japan.^2^

The proportion of elderly people older than 65 years would reach more than 30% by 2030 in Japan,^3^ and this may cause one of the big issues in emergency department (ED) such as overcrowding.^4^ It has been reported that elderly patients stay longer in ED than younger patients because of their atypical reactions or social background,^5^ and so we have to make proper and quick decision at ED.

Several risk‐scoring systems have been established to identify the risk of death in ED. The National Early Warning Score (NEWS) is the most famous and was developed in 2012 in the United Kingdom by the National Early Warning Score Development and Implementation Group on behalf of the Royal College of Physicians.^6^

A previous study showed that NEWS had moderate value for predicting mortality in elderly patients in the ED setting,^7^ and therefore, we need more a powerful triage tool for predicting elderly patient's mortality.

Soluble urokinase plasminogen activator receptor (suPAR) is a nonspecific inflammatory biomarker that reflects the risk of mortality in the general populations.^8^, ^9^, ^10^, ^11^ Several studies have revealed that modified suPAR score with the NEWS or other triage scores improved its ability to predict mortality.^12^, ^13^ However, most studies on suPAR were carried out in Denmark,^13^, ^14^ and no studies focused on the predictive ability of suPAR for mortality among elderly patients in Japan. As a result, the real ability of suPAR and modified suPAR with NEWS for detecting mortality in elderly emergency patients also remains unknown.

The aim of this study is to evaluate the ability of suPAR and modified suPAR with NEWS for detecting mortality in elderly emergency patients who are older than 70 years.

## METHODS

### Study design

This is a secondary analysis of our previous study, which was a single‐center prospective pilot study, carried out for 21 months in the ED of a secondary emergency institution in Japan.

The protocol for this research project was approved by a suitably constituted Ethics Committee of the institution and conforms to the provision of the Declaration of Helsinki [Committee of Jikei University School of Medicine, Approval No. 32‐066 (10141)/Committee of Association of EISEIKAI Medical and Healthcare Corporation Minamitama Hospital, Approval No. 2020‐Ack‐04].

### Study setting and population

This study was carried out between September 16, 2020, and June 21, 2022 at the Association of EISEIKAI Medical and Healthcare Corporation Minamitama Hospital, a secondary emergency institution with 170 beds. The hospital is located in Hachioji City in Tokyo prefecture. The population of Hachioji City is 562,145, with 124,038 (22.07%) of the population being older than 70 years.^15^ All the nontraumatic emergency patients aged 70 years or older were included in this study. They came to the ED either on a Fire Department Ambulance, elder‐care and welfare taxi, or their own car. Exclusion criteria were those who did not consent to the study, and in this study, all participants agreed, and so there were no excluded cases.

### Data sources and measurements

We obtained the clinical information or laboratory sample at the time of patient's arrival to the ED as soon as possible. The suPAR was measured using a suPARnostic Quick Triage and suPARnostic aLF Quick Test Reader (ViroGates A/S, Birkerød, Denmark). The procedure was as follows: (1) 100 μL of running buffer was transferred to an empty tube; (2) 10 μL of plasma sample was transferred to a tube containing 100 μL of running buffer and a pipette was used to mix the sample up and down; (3) then 60 μL of the diluted sample was transferred to the well of the suPARnostic Quick Triage device; (4) and the device was incubated for 20 min on the table, inserted into the aLF Reader, and read.

Complete blood cell counts, serum chemistry, and blood coagulation test were also carried out using the collected blood sample.

The NEWS was derived from seven common physiological vital signs: respiratory rate, peripheral oxygen saturation, the presence of inhaled oxygen parameters, body temperature, systolic blood pressure, pulse rate, and AVPU (Alert, responds to Voice, responds to Pain, Unresponsive) score. The scores vary between 0 and 3 for each parameter. The NEWS totals range from 0 to a maximum of 20. We defined the modified suPAR with NEWS as the blood concentration of suPAR (ng/mL) plus NEWS score. The Charlson Comorbidity Index (CCI) is a method of categorizing comorbidities of patients based on the International Classification of Diseases—10th revision (ICD‐10) diagnostic codes. A weighted score is assigned to each of the 17 comorbidity groups and age, and the scores vary between 1 and 6. A score of 0 indicates that no comorbidities were found.^16^ Diagnostic categories were based on the ICD‐10 and classified as (1) Neurology, (2) Pulmonology, (3) Cardiology, (4) Gastroenterology, (5) Endocrinology, (6) Nephrology/Urology, (7) Hematology, (8) Collagen disease, (9) Gynecology, (10) Dermatology, (11) Toxicology, and (12) Others.

The patients were followed up until 28 days after their ED visit. We gathered the information about patient's condition such as death by telephone at 48 h, 7 days, and 28 days after emergency consultation.

### Statistical analysis

Continuous variables were described as medians and interquartile ranges and were compared by Student's *t*‐test and Mann–Whitney *U*‐test. Categorical variables were described as numbers and percentages and were compared by Fisher's exact test. Receiver‐operating characteristic analysis and the area under the receiver‐operating characteristic curve (AUC) were used to evaluate the predictive value of several variables such as suPAR, NEWS, white blood cell count (WBC), C‐reactive protein (CRP), Cr (creatinine), Na (sodium), and age for mortality. Confidence intervals (CIs) around the AUC were calculated using bootstrap resampling methods with 1,000 repetitions using R software (R version 3.5.3 binary for OS X 10.11; EI Capitan; R Foundation). The cut‐off values for the suPAR, NEWS, WBC, CRP, Age, Cr, and Na were determined using Youden's index (sensitivity + specificity − 1). Using these determined cut‐off points, the sensitivity and specificity were calculated for the prediction of mortality. A *P*‐value of less than 0.05 was considered to indicate statistical significance. Calibration was assessed statistically using the Hosmer–Lemeshow *C* statistic. A statistically significant result suggests a lack of calibration. Previous studies showed that the mortality rate of elderly patients varied between the studies and we estimated it to be approximately 15%.^17^, ^18^, ^19^ A sample size of 47 participants was determined based on 80% power, 0.05 significance level, five allocation ratio, and 0.8 expected AUC Data S1. Data were analyzed using SPSS, version 16.0 (IBM Inc, Chicago, IL, USA).

## RESULTS

Tables 1 and 2 show the comparison of clinical and laboratory parameters between the survivors and nonsurvivors, including the total population. During the study period, 47 eligible elderly patients were included in our study. The median age (interquartile range) of the patients was 88.0 (8.5) years, and 11 (23.4%) patients were male. The major comorbidity categories were cardiology (*n* = 35, 74.5%), neurology (*n* = 34, 72.3%), and endocrinology (*n* = 15, 31.9%). The major symptoms were fever (*n* = 29, 61.7%), hypoxia (*n* = 24, 51.1%), and shortness‐of‐breath (*n* = 15, 31.9%). The major diagnostic categories were pulmonology (*n* = 12, 25.5%), nephrology (*n* = 10, 21.3%), and other cases (*n* = 8, 17.0%); 3 (6.4%) patients died within 48 h, 4 (8.5%) patients died within 7 days, and 8 (17.0%) patients died within 28 days of presenting to the ED. The cause of death was pneumonia (*n* = 5, 62.5%), heart failure (*n* = 3, 37.5%), urinary tract infection (*n* = 1, 12.5%), colon cancer (*n* = 1, 12.5%), and multiple organ failure (*n* = 1, 12.5%); moreover, the cause of death was almost similar with the diagnosis for admission. The median (interquartile range) suPAR and NEWS were 9.0 ng/mL (5.6) and 4 ng/mL (4.0), respectively.

**Table 1:** Comparison of clinical parameters between the survivors and nonsurvivors

	Median (interquartile range)			*P‐*value
	Total population (*n* = 47)	Survivors (*n* = 39)	Nonsurvivors (*n* = 8)	
Age (years), median (IQR)	88.0 (8.5)	87.0 (9.5)	92.0 (8.0)	0.10
Sex, *n* (%)				
Male	11 (23.4)	9 (23.1)	2 (25.0)	>0.99
Female	36 (76.6)	30 (76.9)	6 (75.0)	
Height (m), median (IQR)	1.5 (0.12)	1.5 (0.1)	1.5 (0.1)	0.42
Body weight (kg), median (IQR)	41.0 (14.0)	41.0 (19.0)	41.9 (6.5)	0.86
Body mass index (kg/m^2^), median (IQR)	19.1 (3.4)	19.1 (4.0)	19.5 (3.5)	0.89
The level of care needed, *n* (%)				
Independence	7 (14.9)	7 (17.9)	0 (0.0)	0.66
Requiring help 1	2 (4.3)	2 (5.1)	0 (0.0)	
Requiring help 2	2 (4.3)	1 (2.6)	1 (12.5)	
Long‐term care level 1	5 (10.6)	5 (12.8)	0 (0.0)	
Long‐term care level 2	8 (17.0)	7 (17.9)	1 (12.5)	
Long‐term care level 3	9 (19.1)	7 (17.9)	2 (25.0)	
Long‐term care level 4	4 (8.5)	3 (7.7)	1 (12.5)	
Long‐term care level 5	10 (21.3)	7 (17.9)	3 (37.5)	
Visiting route, *n* (%)				
Facility	24 (51.1)	19 (48.7)	5 (62.5)	0.70
Home	23 (48.9)	20 (51.3)	3 (37.5)	
Comorbidity, *n* (%)				
Pulmonology	8 (17.0)	6 (15.4)	2 (25.0)	0.80
Cardiology	35 (74.5)	28 (71.8)	7 (87.5)	
Neurology	34 (72.3)	27 (69.2)	7 (87.5)	
Gastroenterology	9 (19.1)	9 (23.1)	0 (0.0)	
Endocrinology	15 (31.9)	13 (33.3)	2 (25.0)	
Nephrology	5 (10.6)	5 (12.8)	0 (0.0)	
Collagen disease	1 (2.1)	1 (2.6)	0 (0.0)	
Cancer	14 (29.8)	11 (28.2)	3 (37.5)	
Charlson Comorbidity Index, median (IQR)	6.0 (2.0)	6.0 (2.0)	7.0 (3.0)	0.34
Symptoms of chief complain, *n* (%)				
Fever	29 (61.7)	25 (64.1)	4 (50.0)	0.75
Shock	8 (17.0)	5 (12.8)	3 (37.5)	
Hypoxia	24 (51.1)	18 (46.2)	6 (75.0)	
Cough	10 (21.3)	8 (20.5)	2 (25.0)	
Shortness of breath	15 (31.9)	12 (30.8)	3 (37.5)	
Stomachache	5 (10.6)	4 (10.3)	1 (12.5)	
Nausea/Vomit	3 (6.4)	2 (5.1)	1 (12.5)	
Bloody stool	1 (2.1)	1 (2.6)	0 (0.0)	
Diarrhea	2 (4.3)	0 (0.0)	2 (25.0)	
Convulsion	1 (2.1)	1 (2.6)	0 (0.0)	
Syncope	1 (2.1)	1 (2.6)	0 (0.0)	
Disturbance of consciousness	11 (23.4)	9 (23.1)	2 (25.0)	
Chest pain	2 (4.3)	2 (5.1)	0 (0.0)	
Fatigue	8 (17.0)	7 (17.9)	1 (12.5)	
Weakness	9 (19.1)	7 (17.9)	2 (25.0)	
Loss of appetite	14 (29.8)	12 (30.8)	2 (25.0)	
Body aches	1 (2.1)	1 (2.6)	0 (0.0)	
Diagnostic category, *n* (%)				
Pulmonology	12 (25.5)	10 (25.6)	2 (25.0)	0.28
Cardiology	6 (12.8)	3 (7.7)	3 (37.5)	
Neurology	2 (4.3)	2 (5.1)	0 (0.0)	
Gastroenterology	7 (14.9)	5 (12.8)	2 (25.0)	
Endocrinology	2 (4.3)	2 (5.1)	0 (0.0)	
Nephrology	10 (21.3)	9 (23.1)	1 (12.5)	
Others	8 (17.0)	8 (20.5)	0 (0.0)	

Data are presented as the median (IQR) for continuous variables and *n* (%) for categorical variables. Age and Height were compared by Student's *t*‐test, and the other continuous variables were compared by the Mann–Whitney *U*‐test.

IQR, interquartile range.

**Table 2:** Comparison of laboratory parameters between the survivors and nonsurvivors

Parameters	Median (interquartile range)			*P‐*value
	Total population (*n* = 47)	Survivors (*n* = 39)	Nonsurvivors (*n* = 8)	
Laboratory test, median (IQR)				
WBC (×10^3^/μL)	7.7 (5.6)	7.7 (5.8)	10.3 (4.6)	0.16
Hb (g/dL)	11.3 (2.9)	11.4 (2.4)	10.5 (3.8)	0.95
PLT (×10^4^/μL)	20.6 (8.8)	21.3 (9.4)	17.0 (7.3)	0.08
TP (g/dL)	6.8 (1.1)	6.8 (1.2)	6.6 (0.6)	0.89
Alb (g/dL)	3.2 (0.7)	3.3 (0.8)	3.1 (0.4)	0.72
AST (U/L)	26.0 (22.0)	24.0 (13.5)	45.5 (34.8)	0.12
ALT (U/L)	17.0 (19.0)	16.0 (15.5)	26.0 (26.0)	0.16
LDH (U/L)	246.0 (126.0)	240.0 (121.0)	332.5 (355.3)	0.05
ChE (U/L)	196.0 (102.5)	197.0 (103.0)	162.0 (85.0)	0.41
CK (U/L)	58.0 (90.5)	55.0 (67.0)	125.5 (207.3)	0.06
T‐Bil (mg/dL)	0.8 (0.5)	0.8 (0.6)	0.9 (0.2)	0.75
ALP (U/L)	268.0 (133.5)	265.0 (121.5)	313.5 (154.0)	0.22
γ‐GT (U/L)	21.0 (23.5)	19.0 (19.0)	32.5 (73.0)	0.39
BUN (mg/dL)	24.0 (17.5)	21.0 (19.0)	29.0 (7.8)	0.08
Cr (mg/dL)	0.87 (0.53)	0.9 (0.4)	1.3 (1.2)	<0.01
Na (mEq/L)	138.0 (7.0)	137.0 (6.0)	142.0 (2.3)	<0.05
K (mEq/L)	4.2 (0.9)	4.3 (0.9)	4.2 (0.5)	0.57
Cl (mEq/L)	100.0 (8.0)	100.0 (7.5)	102.0 (5.3)	0.15
CRP (mg/dL)	3.22 (8.8)	2.5 (8.9)	5.9 (3.3)	0.47
PT (%)	80.8 (19.4)	78.0 (20.3)	86.8 (9.8)	0.40
APTT (s)	31.9 (6.9)	31.9 (6.6)	31.9 (5.3)	0.79
Fib (mg/dL)	334.0 (124.0)	344.0 (151.0)	330.5 (75.3)	0.16
Disposition, n (%)				
Discharge	8 (17.0)	8 (20.5)	0.0 (0.0)	<0.001
Admission to a ward	37 (78.7)	29 (74.4)	8 (100.0)	
Admission to high care unit	2 (4.3)	2 (5.1)	0 (0.0)	
suPAR (ng/mL), median (IQR)	9.0 (5.6)	8.8 (4.8)	14.7 (3.5)	<0.01
NEWS, median (IQR)	4.0 (4.0)	4.0 (4.0)	7.5 (3.3)	<0.01

Data are presented as the median (interquartile range) for continuous variables and *n* (%) for categorical variables. Hb, PLT, TP, Alb, K, and Fib were compared by Student's *t*‐test, and the other continuous variables were compared by the Mann–Whitney *U*‐test. Alb, albumin; ALP, alkaline phosphatase; ALT, alanine aminotransferase; APTT, activated partial thromboplastin time; AST, aspartate aminotransferase; BUN, blood urea nitrogen; ChE, cholinesterase; CK, creatinine phosphokinase; Cl, chlorine; Cr, creatinine; CRP, C‐reactive protein; Fib, fibrinogen; Hb, hemoglobin; K, potassium; LDH, lactate dehydrogenase; Na, sodium; NEWS, National Early Warning Score; PLT, platelets; PT, prothrombin time; suPAR, soluble urokinase plasminogen activator receptor; T‐Bil, total bilirubin; TP, total protein; WBC, white blood cell count; γ‐GT, γ‐glutamyl transpeptidase.

There was no significant difference between the survivors and nonsurvivors for any of the clinical parameters. Cr and Na were significantly higher in the nonsurvivor's group than in the survivor's group (*P* < 0.01 and *P* < 0.05, respectively), whereas there was no significant difference between the survivors and nonsurvivors for inflammatory markers such as WBC or CRP.

Soluble urokinase plasminogen activator receptor and NEWS were significantly higher in the nonsurvivor's group than in the survivor's group (*P* < 0.01 and *P* < 0.01, respectively).

**Fig. 1:**
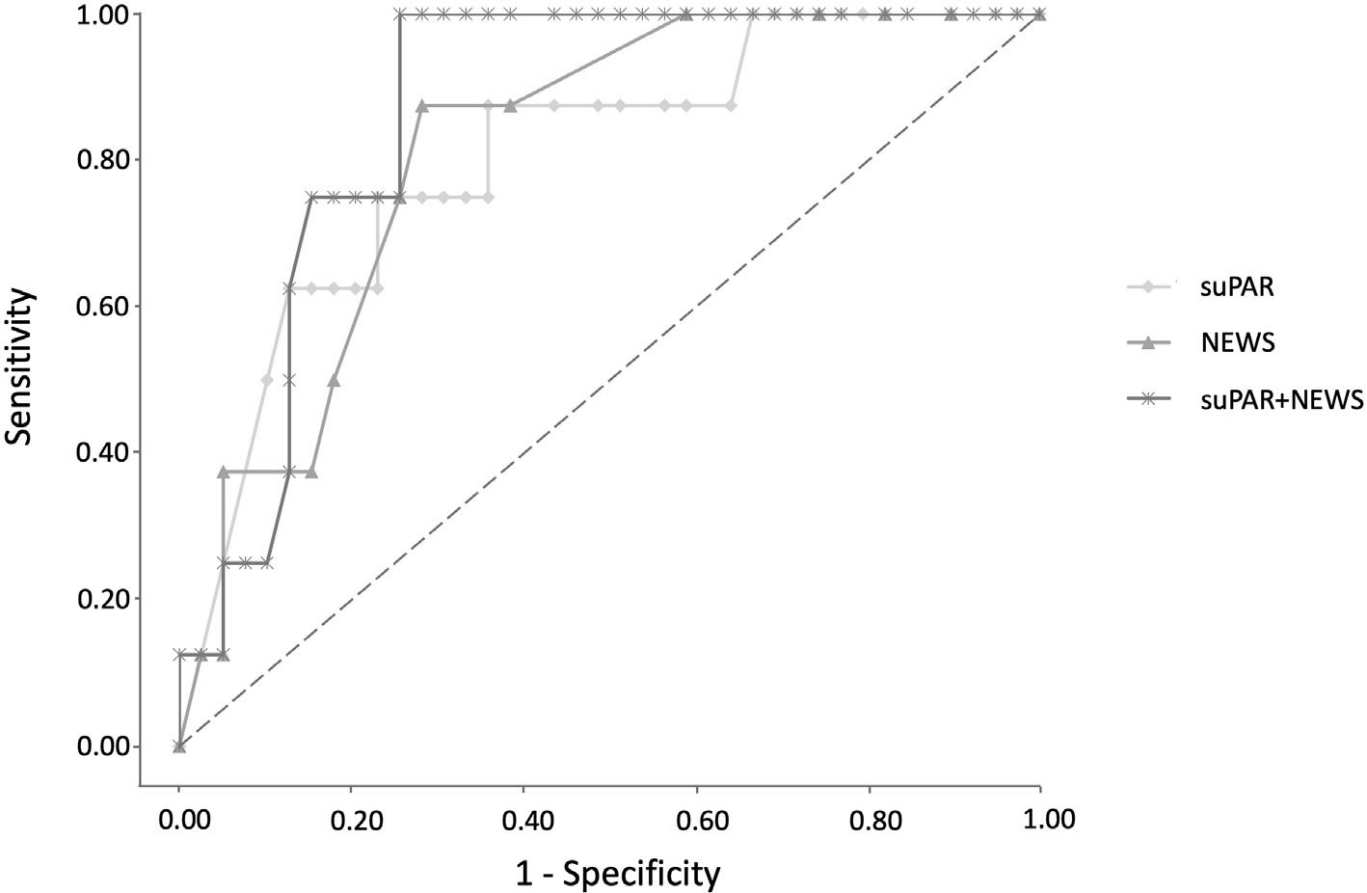
Receiver‐operating characteristic curves for mortality comparing suPAR, NEWS, and suPAR + NEWS in the emergency department. NEWS, National Early Warning Score; suPAR, soluble urokinase plasminogen activator receptor.

Table 3 and Figure 1 show the receiver‐operating characteristic analysis and Hosmer–Lemeshow fit test for the prediction of mortality. The AUC of suPAR was almost the same as that of NEWS. The AUC for predicting mortality was 0.805 (95% CI 0.633–0.949, *P* < 0.001) for suPAR, 0.816 (95% CI 0.668–0.934, *P* < 0.001) for NEWS, 0.792 (95% CI 0.611–0.912, *P* < 0.001) for Cr, 0.756 (95% CI 0.572–0.923, *P* < 0.01) for Na, 0.660 (95% CI 0.474–0.824, *P* = 0.096) for WBC, 0.582 (95% CI 0.397–0.758, *P* = 0.402) for CRP, and 0.644 (95% CI 0.444–0.840, *P* = 0.165) for age. Modified suPAR with NEWS improved the predictive value for mortality, and the AUC for predicting mortality was 0.865 (95% CI 0.761–0.970, *P* < 0.001). However, there was no significant difference between modified suPAR and original suPAR or NEWS. The cutoff values for mortality were 12.2 for suPAR, 6 for NEWS, and 17.5 for suPAR + NEWS. All scores were well calibrated for predicting mortality.

**Table 3:** Receiver‐operating characteristic curve analysis and Hosmer–Lemeshow fit test for the prediction of mortality

	Area under the receiver‐operating characteristic curve (95% CI)	*P*‐value	Cut‐off values	Sensitivity (%)	Specificity (%)	Hosmer–Lemeshow *C* statistic (chi‐square)
1. suPAR	0.805 (0.633–0.949)	<0.001	12.2	75.0	76.9	7.09
2. NEWS	0.816 (0.668–0.934)	<0.001	6	87.5	71.8	6.42
3. suPAR + NEWS	0.865 (0.747–0.958)	<0.001	17.5	100.0	74.4	5.37
4. WBC	0.660 (0.474–0.824)	0.096	10.1	62.5	66.7	15.07
5. CRP	0.582 (0.397–0.758)	0.402	5.01	75.0	64.1	13.35
6. Age	0.644 (0.444–0.840)	0.165	91	62.5	64.1	3.35
7. Cr	0.792 (0.611–0.912)	<0.001	0.94	87.5	66.7	7.08
8. Na	0.756 (0.572–0.923)	<0.01	140	87.5	69.2	13.78

CI, confidence interval; Cr, creatinine; CRP, C‐reactive protein; Na, sodium; NEWS, National Early Warning Score; suPAR, soluble urokinase plasminogen activator receptor; WBC, white blood cell count.

*P* < 0.05: 2 versus 5, 3 versus 4, 5 versus 7. *P* < 0.01: 1 versus 5, 3 versus 5.

## DISCUSSION

This study demonstrated that suPAR and NEWS were significantly higher in the nonsurvivor's group; moreover, the study revealed the high utility of modified suPAR with NEWS for predicting mortality. This is the first study to evaluate the predictive value of modified suPAR with NEWS for predicting mortality in the elderly population in Japan.

We set this study as a prospective pilot study, and determined the sample size with a power of 80% and an AUC of 0.8. Several studies have shown that the AUC of suPAR for in‐hospital mortality was 0.8–0.9,^11^, ^13^, ^14^ which was almost the same as that of NEWS for in‐hospital mortality.^20^, ^21^ Furthermore, a study carried by Kim *et al*.^22^ showed that the AUC of NEWS for in‐hospital mortality was 0.82 in patients older than 65 years. According to these results, we hypothesized that the AUC of suPAR for mortality in elderly patients was almost the same as that of NEWS; thus, we determined the AUC of the sample size for mortality to be 0.8. In our study, we included eight cases of nonsurvivors and 39 survivors based on the sample size test, and the AUC of suPAR for mortality was 0.805, which was almost the same as the set value of 0.8.

In a study carried by Rafal *et al*.,^23^ the suPAR level of elderly people was higher than that of younger people. In this study, however, there was no significant difference in age between the survivor's group and the nonsurvivors group, and therefore we assumed that the age between the two groups had no effect on the suPAR level.

A study carried by Qu *et al*.^24^ showed that the AUC of WBC and CRP for ICU mortality was low (0.568 and 0.684, respectively). Our study also demonstrated the low utility of WBC and CRP for predicting mortality, and these results were the same as that of the previous study.

A study carried by Rasmussen *et al*.^12^ revealed that the AUC of suPAR combined with age, sex, and NEWS for predicting in‐hospital mortality was 0.92, which is significantly greater than that of suPAR alone (0.84; *P* < 0.0001). By contrast, in our previous study, the AUC of NEWS for mortality in elderly patients was relatively lower than that of studies in general population, and the AUC was 0.789.^7^ According to these results, the AUC of modified suPAR with NEWS in elderly patients for mortality was lower than that of combined suPAR in general population.

Several studies have already revealed that high plasma suPAR levels have been associated with increased severity and mortality in coronavirus disease 2019 (COVID‐19),^25^ sepsis,^26^ cancer,^27^ cardiovascular diseases,^28^ and type 2 diabetes mellitus.^29^ In the present study, CCI and comorbidities were not significantly different between survivors and nonsurvivors, and therefore we suspected that suPAR concentration would not also be affected by CCI or comorbidities but reflects the overall condition of the patients.

This study has several limitations. First, this was a pilot study and we included a small number of elderly patients. Second, we had to gather the cases under the COVID‐19 pandemic, so there may be selection bias in the study population. Third, our hospital is a secondary emergency institution, and so we did not treat very fatal cases, which could be another cause of selection bias.

## CONCLUSION

Our single‐center study has demonstrated the high utility of modified suPAR with NEWS as a predictive tool of mortality in elderly emergency patients. Evidence from multicenter studies is needed for introducing modified suPAR with NEWS in the ED setting.

## DISCLOSURE

Approval of the Research Protocol with Approval No. and Committee Name: The study was approved by a suitably constituted Ethics Committee of the institution and conforms to the provision of the Declaration of Helsinki (Committee of Jikei University School of Medicine, Approval No. 32‐066 (10141)/Committee of Association of EISEIKAI Medical and Healthcare Corporation Minamitama Hospital, Approval No. 2020‐Ack‐04).

Informed Consent: The written consent was obtained from all the participants.

Registry and the Registration No. of the Study/Trial: N/A.

Animal Studies: N/A.

Conflict of Interest: None declared.

## Supporting information




**Data S1** The master sheet contains physical and laboratory parameters other than vital signs, whereas the EWS sheet contains the vital signs and NEWS score.
